# Alcohol’s Contribution to Compromised Immunity

**Published:** 1997

**Authors:** Gyongyi Szabo

**Affiliations:** Gyongyi Szabo, M.D., Ph.D., is a research associate professor of medicine in the Department of Medicine, University of Massachusetts Medical Center, Worcester, Massachusetts

**Keywords:** immune system, immune disorder, immune response, AODE (alcohol and other drug effects), cytokines, inflammation, oxygen radicals, bacterial disease, HIV infection, trauma, njury, pathologic process, literature review

## Abstract

Alcoholics frequently suffer from infectious diseases and have increased rates of some cancers, indicating that alcohol impairs the immune system, which protects the body against this type of damage. Alcohol interferes with the functions of many of the cells and molecules that are part of the immune system. For example, alcohol inhibits the functions of the cells that ingest and destroy invading microorganisms (i.e., neutrophils, monocytes, and macrophages). Both acute and chronic alcohol exposure also alter the production of signaling molecules that help coordinate the immune response (i.e., cytokines). Finally, alcohol adversely affects the functions of the cells that mediate the immune response against specific microorganisms and long-term immunity (i.e., T cells and B cells). As a result, alcoholics have an increased susceptibility to diseases caused by bacterial infections, such as tuberculosis and pneumonia. Alcoholics also may be more susceptible to infections from the virus that causes AIDS. In addition, alcohol intoxication can exacerbate the immune suppression that occurs after traumatic injuries.

The immune system serves as the body’s defense against infections by microorganisms; damage caused by other foreign substances; and the uncontrolled, tumorous growth of the body’s own cells. Impairment of this system can increase a person’s risk for developing various illnesses, including infectious diseases, such as tuberculosis, and certain types of cancer. Alcohol can modulate this defense, and clinicians have known for a long time that chronic alcohol abusers have an impaired immune system. This impairment manifests itself in several ways. For example, alcoholics are prone to infections by various disease-causing microorganisms (i.e., pathogens); have a decreased ability to fight these infections; and have an increased risk of developing tumors, particularly in the head, neck, and upper gastrointestinal tract. Although alcohol-induced malnutrition—including vitamin deficiencies—and advanced liver cirrhosis likely contribute to some abnormalities in the immune system of alcoholics, alcohol itself also is a potent modulator of immune functions. Interestingly, not only chronic alcohol abuse but also single-episode (i.e., acute) and/or moderate alcohol consumption can affect the immune system.

This article briefly reviews the main features and components of the immune system and summarizes some of the consequences and mechanisms of alcohol use on the body’s defense against pathogens.

## The Immune System—An Overview

The immune system has two main arms: innate, or nonspecific, immunity and acquired, or specific, immunity. Innate immunity exists before the body is exposed to a pathogen for the first time. Moreover, this system does not respond to specific pathogens but instead responds to any pathogen it encounters. For example, the cells involved in innate immunity immediately attack any kind of bacterium or virus that enters the body, whether it is the first or second infection by that organism. Acquired immunity, in contrast, is activated only after the body is exposed to a pathogen for the first time. In addition, the acquired response is specific to one particular pathogen. For example, when *Mycobacterium tuberculosis*, the bacterium that causes tuberculosis, enters the body, the contact with that pathogen activates cells involved in acquired immunity. These activated cells attack only *M. tuberculosis* and no other bacteria or viruses. The activated cells also generate a kind of immune “memory” that allows the body to fight a second infection by the same pathogen even faster and more efficiently.

The elements of innate immunity include white blood cells that ingest and destroy microorganisms (i.e., phagocytes); certain proteins that circulate in the blood, called the complement^1^ system; and signaling molecules (i.e., cytokines) that are produced and secreted by some of the phagocytes. Several different types of phagocytes exist, with specific functions as follows:

- Neutrophils ingest and thereby destroy pathogens, primarily invading bacteria.
- Monocytes that circulate in the blood or that have entered the tissues (i.e., macrophages) ingest and destroy a variety of foreign substances and microorganisms. Monocytes also exhibit pathogen-derived proteins and other molecules (i.e., antigens) on their surfaces in order to activate other cells in the immune system. Finally, monocytes and macrophages secrete cytokines that help regulate immune system activity.
- Natural killer (NK) cells recognize and eliminate cells in the body that have been infected by parasites or that have turned into cancer cells.

The elements of acquired immunity include numerous cell types and molecules that function cooperatively to mount a complex host defense and thereby amplify and focus the protection offered by the innate immunity. The most important cells involved in acquired immunity are T lymphocytes, or T cells, and B lymphocytes, or B cells. These cells circulate in the blood or reside in special lymphoid tissues (e.g., the spleen, lymph nodes, and tonsils), where they can encounter antigens and initiate an immune response.

T cells and B cells are the cornerstones of two types of immune responses, the cell-mediated immunity and the antibody-mediated (i.e., humoral) immunity. The cell-mediated immunity relies primarily on T cells that are activated by exposure to antigen-presenting cells (e.g., monocytes, macrophages, and B cells). Each antigen-presenting cell displays only one antigen (e.g., a viral protein) on its surface and thus stimulates only T cells that recognize this specific antigen. The activated T cell then can bind to other cells carrying the same antigen (e.g., virus-infected cells) and initiate their destruction. Several sub-populations of T cells have specific functions in the complex chain of events occurring during an immune response:

- Helper T cells produce and secrete cytokines that stimulate the activity of other immune cells.
- Cytotoxic T cells recognize antigens on the surface of virus-infected or transplanted cells and destroy these cells.
- Suppressor T cells inhibit other immune responses, thereby preventing overreaction of the immune system.
- Delayed-type hypersensitivity T cells produce cytokines that induce a localized inflammatory response and attract macrophages and cytotoxic T cells to that site to eliminate the antigen.

The B cells produce the humoral immunity. These cells carry immune proteins (i.e., antibodies, or immunoglobulins) on their surface that recognize and bind to antigens. Like T cells, each B cell also recognizes only one specific antigen and becomes activated when it comes into contact with it. Most activated B cells develop into so-called plasma cells, which secrete their antibodies into the blood or lymph. There the antibodies can bind to their target antigens (e.g., a virus or a virus-infected cell) and thus mark them for destruction. Other B cells become memory cells, which help the body fight a second infection by the same pathogen more expeditiously.

The T-cell and B-cell responses are not independent of each other, however, but are intricately intertwined. Thus, B cells that have bound an antigen serve as antigen-presenting cells that can activate a T-cell response. Moreover, B cells and T cells communicate with each other and with other immune cells by secreting numerous cytokines that can influence various components of both the nonspecific and specific immune responses. For example, some T cells produce cytokines that stimulate their own activity or that of other T cells. Other subgroups of T cells secrete cytokines that inhibit the cell-mediated and humoral immunity and thus prevent an excessive reaction of the immune system. Finally, some T-cell–derived cytokines enhance B-cell multiplication, differentiation, and antibody production. The largest family of cytokines are the interleukins (IL’s), which are produced in various cell types and have numerous functions (see table).

The following example may help illustrate some of the complex interactions that take place during an immune response. When a person sustains a small injury, such as a cut, bacteria can enter the body and the bloodstream through the wound. Phagocytes (e.g., monocytes and neutrophils) patrolling the blood encounter some of these bacteria; identify them as foreign to the body; and engulf, ingest, and destroy them. During the intracellular breakdown of the ingested bacteria, the phagocytes generate small proteins or protein fragments that serve as antigens. The phagocytes display these antigens on their cell surface, together with certain of their own proteins known as major histocompatibility complex (MHC) proteins. In addition to the phagocytes, proteins of the complement system also recognize the invading bacteria and bind to proteins on the bacterial surface. This binding triggers several biochemical processes that eventually lead to the destruction of the bacteria.

**Table:** Important Cytokines of the Immune System

Cytokine	Primary Source	Principal Functions
*Inflammatory cytokines*		
Interferon alpha (IFN-α)	Macrophages	Induces protection against viral infections; activates macrophages; and inhibits the growth of various cell types
Interleukin 1 (IL-1)	Macrophages and other cells	Produces inflammatory responses; induces fever; stimulates proliferation of helper T cells; and promotes B-cell growth and differentiation
Interleukin 6 (IL-6)	Macrophages, T cells, and other cells	Promotes maturation of stimulated B cells to antibody-secreting plasma cells; acts with other cytokines to stimulate immature and mature T cells; and stimulates production of complement factors and other mediators of inflammatory responses
Tumor necrosis factor alpha (TNF-α)	Macrophages, T cells, natural killer (NK) cells, and other cells	Promotes inflammatory responses; stimulates neutrophils and macrophages; induces fever; and induces macrophages to produce IL-1, IL-6, and TNF-α

*Immunoregulatory cytokines*		
Interleukin 10 (IL-10)	Macrophages and T cells	Inhibits T-cell proliferation; reduces production of inflammatory cytokines; and promotes B-cell proliferation and antibody secretion
Interleukin 12 (IL-12)	Monocytes and macrophages	Activates NK cells; activates a subtype of T cells (i.e., CD4 T cells); and induces the cell- mediated (i.e., Th1) immune response
Transforming growth factor beta (TGF-β)	Macrophages and T cells	Inhibits T-cell proliferation; reduces production of inflammatory cytokines; augments B-cell proliferation and antibody secretion; and promotes collagen production

*Chemokines*		
Interleukin 8 (IL-8)	Macrophages	Attracts neutrophils to the site of an infection

*Lymphokines*		
Interferon gamma (IFN-γ)	T cells	Induces protection against viral infection; stimulates macrophages and neutrophils; enhances expression of major histocompatibility complex (MHC) proteins on many cells; and promotes B-cell and T-cell differentiation
Interleukin 2 (IL-2)	T cells	Stimulates proliferation of T cells; enhances activity of NK cells; and stimulates B-cell proliferation and antibody production
Interleukin 4 (IL-4)	A subtype of T cells (i.e., CD4 T cells)	Stimulates T-cell growth; induces B-cell activation and growth; and modulates antibody production by B cells

T cells circulating in the blood recognize phagocytes simultaneously displaying antigens and MHC proteins. The T cells bind to the phagocyte-bound antigens through the help of docking molecules, called T-cell receptors. The activated T cells multiply and begin secreting cytokines, which, in turn, activate cytotoxic T cells that can then recognize, bind to, and destroy cells infected by the invading bacteria.

Parallel to the T-cell response, the B cells mount another line of defense against the invading bacteria. Thus, antibodies on the B-cell surface recognize and bind to antigens on the bacterial surface. This binding activates the B cells, which then differentiate into plasma cells that secrete large amounts of antibodies. The antibodies are distributed throughout the bloodstream and bind to the bacteria wherever they encounter them, aided by proteins of the complement system. The antibody-covered bacteria clump together and become new targets for monocytes and other phagocytes.

## Alcohol’s Effects on the Immune Defense

The body’s response to an invading pathogen can be divided into two phases. The first phase is an inflammatory reaction, which protects the body from the immediate effects of the infection. The inflammatory response primarily involves phagocytic cells that help eliminate the pathogen, cytokines secreted mainly by these phagocytes, and other molecules (e.g., oxygen radicals) that assist in killing the pathogen. The second phase, the development of immunity to the pathogen, is mediated by T cells and B cells. Alcohol can interfere with both phases of the immune response.

### Alcohol’s Effects on the Inflammatory Response

#### Effects on Phagocytic Cells

During an inflammatory response, chemical substances released by cells at the site of the infection induce phagocytes to migrate from their normal locations in the bloodstream or the tissues to the site of the inflammation. This process is called chemotaxis. Various substances can serve as chemotactic agents, including activated components of the complement system, a group of white blood cell-derived proteins called leukotrienes, and other proteins produced by immune cells (i.e., chemokines, such as interleukin-8 [IL-8]). The neutrophils and monocytes recruited from the bloodstream must adhere to and migrate through the cell layer lining the blood vessels at the site of the infection, ingest the microorganisms, and destroy the ingested pathogens using specific enzymes or toxic, oxygen-derived free radicals.

Alcohol can affect this sequence of events at several levels. Alcohol’s effects on phagocyte adhesion to the blood-vessel walls and chemotaxis were studied in chronically alcohol-fed rats. Neutrophils from these animals exhibited increased levels of CD18 molecules on their surface, which are required for adhesion. Furthermore, chemokines (e.g., IL-8) secreted by macrophages residing in the liver (i.e., Kupffer cells) of the alcohol-fed rats increased the chemotaxis of normal neutrophils. Increased infiltration of neutrophils into the liver, in turn, can lead to liver injury. Thus, an alcohol-induced increase in chemokine production by Kupffer cells may lead to neutrophil accumulation and thus to liver damage (e.g., alcoholic hepatitis). This hypothesis is consistent with observations that in patients with acute alcoholic hepatitis, IL-8 levels in the blood are elevated and may be associated with neutrophil accumulation in the liver.

Increased neutrophil chemotaxis even occurred in rats that received a one-time alcohol injection. In contrast, Kupffer cell chemotaxis decreased under the same conditions. These results suggest that alcohol can differentially affect the functions of various phagocytic cell types. Studies with cultured human cells, however, demonstrated decreased neutrophil chemotaxis after the cells were exposed to alcohol.

Chronic as well as acute alcohol consumption also reduces the ability of phagocytes to ingest and break down pathogenic bacteria. For example, cultured human monocytes exposed to alcohol showed reduced phagocytic functions; moreover, the cells produced less of a receptor protein that is required for the ingestion of antibody-coated particles. In mice, both short-term and long-term alcohol feeding reduced the phagocytic ability of macrophages residing in the membrane lining the abdominal cavity. Thus, abnormal neutrophil adherence and chemotaxis, as well as reduced phagocytic function of macrophages, may contribute to the impaired defense against microorganisms observed after alcohol consumption.

### Effects on Inflammatory Cytokines

Phagocyte contact with pathogens induces the release of cytokines by the phagocytes that help initiate and maintain the inflammatory response and thus play a pivotal role in the body’s immune defense. The most common inflammatory cytokines—tumor necrosis factor alpha (TNF-α), IL-1, and IL-6—are primarily produced by monocytes and macrophages (see figure). During an overwhelming inflammatory response, however, neutrophils, lymphocytes, and other tissue cells also can be sources of inflammatory cytokines. Excessive levels of these cytokines may cause tissue damage, whereas reduced levels may result in an insufficient immune response.

In chronic alcohol abusers, particularly those with alcoholic liver disease, the levels of TNF-α, IL-1, and IL-6 in the blood are significantly elevated. These increased cytokine levels may contribute to most of the signs and symptoms observed in patients with alcoholic hepatitis (e.g., generally increased metabolism, fever, weight loss, elevated levels of proteins produced in the liver, and markers of malnutrition). It is unknown, however, which cells cause the elevated inflammatory cytokine production in alcoholics.

**Figure:**
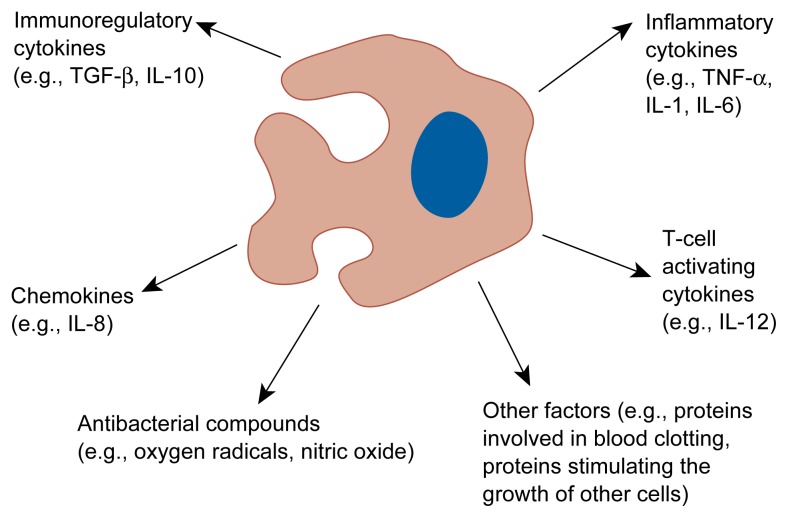
Monocyte/macrophage-derived substances potentially affected by alcohol. Monocytes and macrophages produce numerous substances that initiate and regulate inflammatory reactions; attract other immune cells (i.e., chemokines); stimulate T cells; help in the elimination of pathogens, such as bacteria; and perform other functions throughout the body. Alcohol may interfere with the production and secretion of all these substances, thereby impairing the body’s immune response. IL = interleukin; TGF-β = transforming growth factor beta; TNF-α = tumor necrosis factor alpha.

Acute, moderate alcohol consumption, in contrast, transiently reduces the pathogen-induced production of inflammatory cytokines. For example, cultured human monocytes exposed to alcohol before being stimulated with various bacterial antigens produced lower-than-normal levels of TNF-α, IL-1, and IL-6. Similarly, the TNF-α levels produced in response to a challenge with a bacterial antigen were decreased in mice that had received a single dose of alcohol. Considering the pivotal role of TNF-α in the defense against microorganisms, impaired inflammatory cytokine production after acute alcohol exposure significantly compromises the body’s defense system.

#### Effects on Immunoregulatory Cytokines

The initial inflammatory response to pathogens normally is turned off by regulatory cytokines whose production typically is induced in a later phase of the infection. The most studied immunoregulatory cytokines are IL-10 and transforming growth factor beta (TGF-β), both of which are produced by macrophages and T cells. IL-10 promotes humoral immunity and inhibits cell-mediated immunity by reducing the production of several cytokines, including inflammatory cytokines, and by preventing the multiplication (i.e., proliferation) of T cells. Acute alcohol exposure increases IL-10 production in cultured human monocytes both in the absence and presence of stimulation by bacterial antigens and thus may interfere with the normal interaction of the cell-mediated and humoral immunities.

The other cytokine controlling inflammatory reactions and T-cell proliferation is TGF-β. In experiments using cultured monocytes, physiologically relevant alcohol concentrations^2^ activated both the baseline and the bacterial antigen-induced TGF-β production. Elevated TGF-β levels may have multiple implications for immune-system functioning, including inhibition of inflammatory cytokine production by monocytes and other cells, inhibition of T-cell proliferation, and augmentation of the humoral immune response. As a result, the drinker becomes more susceptible to infections and exhibits decreased immune system activity in eliminating infections. In addition, elevated TGF-β levels promote collagen production. Collagen molecules normally form the fibers making up tendons and ligaments. However, excessive collagen production resulting from alcohol-induced TGF-β may result in abnormal collagen deposits in the liver that have been implicated in the development of some types of alcoholic liver disease.

Alcohol also increases the production of nonprotein regulatory molecules that inhibit the antigen-presenting capacity of monocytes, inflammatory cytokine production, and T-cell proliferation.

By increasing the levels of these substances as well as of IL-10 and TGF-β, alcohol can interfere with the body’s normal defense against invading microorganisms in two ways: by reducing inflammatory-cytokine production and by inhibiting T-cell proliferation.

### Effects on Oxygen-Radical Production

Oxygen radicals (e.g., superoxide anions and hydrogen peroxide) are unstable oxygen-containing molecules that readily interact with other molecules in a cell. Oxygen radicals produced by macrophages and other phagocytes play a crucial role in destroying micro-organisms, especially in the lungs. Researchers found that macrophages in the lungs of acutely or chronically alcohol-fed rats produced fewer super-oxide anions and less hydrogen peroxide than did macrophages from non-alcohol-exposed rats. Furthermore, the lung macrophages produced and secreted less nitric oxide, another molecule with characteristics and functions similar to those of oxygen radicals. The alcohol-induced decreases in the macrophages’ production of oxygen radicals and nitric oxide could undermine the body’s defense against bacteria. This mechanism could contribute to the high incidence of tuberculosis in alcoholics. (For more information on the association of alcohol use and tuberculosis, see sidebar, pp. 39–41.)

**Alcohol and Susceptibility to Tuberculosis**

Worldwide, tuberculosis (TB) is the leading cause of death from a single infectious agent (Flynn and Bloom 1996). *Mycobacterium tuberculosis*, the agent responsible for TB, is transmitted when a person inhales microscopic airborne particles containing the organism (i.e., “droplet nuclei”) coughed up by someone with active TB disease, although prolonged exposure usually is necessary before an infection becomes established. Initially *M. tuberculosis* attacks the lung, where immune cells (i.e., macrophages and lymphocytes) battle the infection. In the absence of adequate immunity, however, the bacteria ingested by macrophages continue to multiply within these immune cells, and characteristic lesions called tubercles eventually form in the lungs. Among other symptoms, people with active TB develop a bloody cough, fatigue, and difficulty breathing. TB can affect any organ system or develop into systemic infection (i.e., miliary TB) when infected cells spread from the lungs and disseminate through the bloodstream. No effective vaccination against TB currently exists, but 6 to 9 months of treatment with multiple antituberculous drugs will cure most TB patients who complete the course of therapy.

Sociological studies show that TB is more prevalent in low-income, densely populated housing areas—settings often associated with high rates of alcoholism.^1^ Numerous studies also have noted a direct association between alcoholism and pulmonary tuberculosis (Jacobson 1992). Such apparent susceptibility to TB infection among alcoholics, especially those who are homeless or indigent, can be attributed both to biological and to social and behavioral factors.

***Biological Factors***

Although approximately one-third of the world’s population is infected with *M. tuberculosis*, only about 10 percent will experience either acute or reactivated disease (Flynn and Bloom 1996). Clearly, the body’s immune system is quite capable of controlling *M. tuberculosis* in most cases. Any illness or condition associated with an impaired immune system, however, can increase the likelihood of developing active TB. In particular, numerous animal studies have confirmed that the body’s control of *M. tuberculosis* infection resides in its ability to mobilize macrophages and lymphocytes against invading organisms (i.e., cell-mediated immunity) (Dunlap and Briles 1993).

Because alcohol use significantly inhibits cell-mediated immunity, it compromises the body’s immune defense. Thus, alcoholism—along with other diseases associated with impaired cell-mediated immunity (e.g., HIV infection)—increases a person’s susceptibility to active TB infection as well as reactivation of latent disease (Flynn and Bloom 1996). Researchers do not know, however, whether alcohol consumption itself or the liver damage and malnutrition often associated with alcoholism are primarily responsible for the impaired immunity of alcoholics.

Investigators studying mechanisms by which alcohol predisposes people to TB recently have focused special attention on the specific immune system components participating in the body’s defense against *M. tuberculosis* and related mycobacteria. Alcohol use has been found to hinder the body’s antimycobacterial defense on multiple levels, including through impaired macrophage response, altered levels of the proteins that act as intercellular mediators (i.e., cytokines), and a disturbed balance between the two basic types of acquired immunity: cell-mediated immunity and immunity provided by circulating antibodies (i.e., humoral immunity). Each of these interrelated consequences is discussed in the sections that follow.

***Impaired Macrophage Response***. Macrophage activation is considered essential for local containment and destruction of mycobacteria (including *M. tuberculosis*) and other invasive microorganisms. Thus, macrophage activity is the hallmark of resistance to TB (Flynn and Bloom 1996). These immune system cells (along with the monocytes that give rise to them) play a key role in directly “presenting” the chemical identifiers that stimulate an immune response (i.e., the antigens) to lymphocytes in the body’s lymph tissue. In response to antigen presentation, certain lymphocytes (i.e., T lymphocytes) develop into T cells that specifically target the *M. tuberculosis* organism for destruction. These T cells rapidly multiply and circulate throughout the body. Evidence from several laboratories suggests that activation of two types of T cells in particular (i.e., CD4 and CD8 T cells) is important in the control of mycobacterial infection (Flynn et al. 1992; Kaufmann and Flesch 1986; Leveton et al. 1989; Orme and Collins 1983, 1984; Flory et al. 1992).

Although alcohol likely affects many immune system cells, macrophages and monocytes appear to be particularly sensitive to its influences. Both acute and chronic alcohol use may decrease the activation of antigen-specific T cells by inhibiting the macrophages’ capacity to present mycobacterial antigen to lymphocytes (Szabo et al. 1993). Bermudez and Young (1991) have shown that alcohol also enhances the survival of another pathogen (i.e., the *Mycobacterium avium* complex, or MAC^2^) within blood-derived macrophages in people and liver macrophages (i.e., Kupffer cells) in mice. The same study demonstrated an increase in MAC colony counts in the blood, liver, and spleen of alcohol-fed mice compared with controls, suggesting that alcohol use prior to and during MAC infection contributes to dissemination of the disease in the body.

In addition to decreasing the antimycobacterial activity of macrophages, alcohol consumption also reduces macrophage response to immune system modifiers. For example, the cytokines known as tumor necrosis factor alpha (TNF-α) and granulocyte-macrophage colony-stimulating factor (GM-CSF) both have been shown to induce macrophages to inhibit the growth of and destroy mycobacteria. In vitro studies suggest that alcohol impedes the protective effect exerted by these cytokines, however (Bermudez and Young 1991).

***Altered Cytokine Levels***. TNF-α, one of the inflammatory mediators derived primarily from macrophages, plays a major role in antimycobacterial defense (Nelson et al. 1995; Flynn and Bloom 1996). This cytokine directly inhibits mycobacterial growth in vitro, recruits additional inflammatory cells, and induces the action of other antimycobacterial mediators (e.g., nitric oxide and reactive oxygen radicals).

One of the most dramatic effects of both acute and chronic alcohol use is the impaired capacity of monocytes to produce cytokines that trigger inflammation, particularly TNF-α, in response to bacterial or mycobacterial infection. In a study with rats, Nelson and colleagues (1989) found that the immune cells first to be exposed to mycobacteria (i.e., macrophages in the alveoli of the lungs) showed a reduced capacity to manufacture TNF-α following both chronic and acute alcohol consumption, an effect that likely would contribute to a compromised immune defense against TB. Of interest, Denis (1991) found that TNF-α had a beneficial effect on survival when it was infused into mice inoculated with *M. tuberculosis*, suggesting that alcohol’s negative effect on the antimycobacterial activity of macrophages potentially could be overcome.

***Disturbed Cell-Mediated and Humoral Immunity Balance***. Both acute and chronic alcohol use are associated with a shift toward the predominance of humoral immune functions at the expense of cell-mediated functions, as indicated by increased levels of circulating antibodies and decreased T-cell activity. Specifically, acute and chronic alcohol use alter the profile of the T-cell population so that the type of cells known as T-helper-2 (Th2), which are associated with humoral immunity, dominate over those known as T-helper-1 (Th1), which are associated with cell-mediated immunity. One recent hypothesis suggests that this shift comes about through alcohol’s effects on monocytes. Monocytes play a pivotal role in supporting a Th1 immune response in two ways: through their capacity to present antigen to activate T cells and through their manufacture of the important cytokines known as interleukin-12 (IL-12) and interleukin-10 (IL-10). IL-12 enhances the activity of CD4 and CD8 T cells, as well as natural killer cells, and triggers a cell-mediated immune response; elevated levels of IL-10, however, inhibit production of IL-12 and other inflammatory cytokines. In recent studies of isolated human monocytes exposed to alcohol, researchers have observed elevated IL-10 levels, inhibition of IL-12, increased production of other mediators that check the immune system (e.g., transforming growth factor beta [TGF-β]), and a reduced antigen-presenting capacity (Szabo et al. 1993, 1996; Mandrekar et al. 1996). All of these effects imply that acute alcohol consumption impairs a cell-mediated (i.e., Th1-type) immune response and consequently tilts the balance of the immune system toward humoral (i.e., Th2-type) immune functions. Given the importance of cell-mediated immunity in overcoming TB infection, the implications of this shift can be significant for alcoholics exposed to *M. tuberculosis*.

Researchers also have found, however, that the cytokine gamma-interferon (IFN-γ) plays a critical role in determining whether a Th1- or Th2-type response will dominate in alcohol-exposed monocytes. Recent studies showed that the presence of IFN-γ decreased alcohol-induced IL-10 production, thus canceling IL-10’s inhibition of IL-12 and thereby augmenting cell-mediated (i.e., Th1-type) immunity (Mandrekar et al. 1996; Szabo et al. 1996). This finding supports the demonstration by Flynn and Bloom (1996) that IFN-γ is essential to resistance against TB in mice. Vicente-Gutierrez and colleagues (1991) have reported decreased IFN-γ in chronic alcoholics, indicating that suppressed IFN-γ levels in alcoholics likely contribute to an impaired cell-mediated immune response during mycobacterial infection.

Taken together, both acute and chronic alcohol use have been shown to predispose the body to compromised defense against mycobacteria. Although researchers still need to refine our understanding of the exact mechanisms by which alcohol use decreases antimycobacterial defense, the immunosuppressive effects of alcohol on monocyte and macrophage function, cytokine production, and antigen-specific T-cell activation appear to be key factors in the increased susceptibility to TB following alcohol use.

***Social and Behavioral Factors***

For alcoholics, especially those who are indigent or homeless, several social and behavioral factors converge to increase their vulnerability to TB and to hinder their recovery from the disease.

***High-Risk Living Conditions***. Typically, indigent and homeless alcoholics dwell in crowded and impoverished living conditions. Such an environment significantly increases their chances for repeatedly inhaling *M. tuberculosis* droplet nuclei. With prolonged exposure, a person is more likely to acquire an active TB infection and subsequently spread the disease by coughing up more infectious droplets for others to inhale. TB outbreaks have occurred in urban homeless shelters and other densely populated residential settings, such as prisons and nursing homes. Kline and colleagues (1995) even reported an outbreak among regular patrons of a neighborhood bar and speculated that heavy alcohol use and a highly infective source could have been contributing factors.

***Behavioral Issues***. The chaotic lifestyles of most indigent alcoholics tend to delay their seeking medical attention until their illness is fairly severe. Consequently, they often show up at a hospital or clinic with extensive TB infection and tissue destruction. Nevertheless, unless their illness is far advanced, alcoholics theoretically should respond just as well as nonalcoholics to medical therapy. Indigent and homeless alcoholics actually have a poorer prognosis than others with TB, however. The primary reason is not a worse response to medications but a relative lack of cooperation in taking them. The same disorganized life circumstances that delay treatment seeking also impede taking regular doses of medication. In addition, the treatments for TB involve drugs that are potentially toxic to the liver and the nervous system, and alcohol enhances their toxicity. Rather than stop drinking, alcoholics may avoid treatment for TB.

Standard drug therapy for TB currently involves 9 months of taking the medications isoniazid and rifampin or 6 months of taking isoniazid, rifampin, and pyrazinamide. After an initial phase of daily medication, patients can receive drugs twice weekly without compromising effectiveness. (The slow growth cycle of *M. tuberculosis* necessitates a long treatment duration, and multiple drugs thwart the organism’s ability to develop resistance.) Because homeless alcoholics frequently have difficulty adapting to hospitalization, outpatient care is the usual approach to managing their illness. Even so, these patients may not comply well with treatment, either by failing to keep their medical appointments consistently or by not completing therapy. Impoverished alcoholics thus are prone to reactivation of TB, and if their medication use is erratic, a strain of *M. tuberculosis* resistant to standard medication is more likely to develop. Contagious alcoholics then spread resistant TB strains to others.

Alcoholics may be especially unlikely to cooperate with treatment if they perceive medical staff as a threat to their drinking—a likely scenario given the dangers of combining TB medication with alcohol. Caregivers should bear in mind that TB is a serious public health hazard and give first priority to resolving a patient’s TB over addressing his or her alcoholism. By providing alcoholic TB patients with friendly, nonthreatening support, caregivers may improve the chances for complete TB treatment and, possibly, succeed in helping the patient accept the need for alcoholism treatment as well.

— Gyongyi Szabo

ReferencesBermudezLEYoungLSEthanol augments intracellular survival of *Mycobacterium avium* complex and impairs macrophage responses to cytokinesJournal of Infectious Diseases1636128612921991203779410.1093/infdis/163.6.1286DenisMInvolvement of cytokines in determining resistance and acquired immunity in murine tuberculosisJournal of Leukocyte Biology5054955011991174884310.1002/jlb.50.5.495DunlapNEBrilesDEImmunology of tuberculosisMedical Clinics of North America776123512511993823140910.1016/s0025-7125(16)30190-0FloryCHubbardRCollinsFEffects of in vivo T lymphocyte subset depletion on mycobacterial infections in miceJournal of Leukocyte Biology512252291992134731110.1002/jlb.51.3.225FlynnJLBloomBRRole of T1 and T2 cytokines in the response toMycobacterium tuberculosis. Annals of the New York Academy of Sciences7951371461996895892410.1111/j.1749-6632.1996.tb52662.xFlynnJLGoldsteinMMTrieboldKJKollerBBloomBRMajor histocompatibility complex class I-restricted T cells are required for resistance to *Mycobacterium tuberculosis* infectionProceedings of the National Academy of Sciences USA891201312017199210.1073/pnas.89.24.12013PMC506881465432JacobsonJMAlcoholism and tuberculosisAlcohol Health & Research World16139451992KaufmannSHEFleschIFunction and antigen recognition pattern of L3T4+ T cell clones from *Mycobacterium tuberculosis-*immune miceInfection and Immunity542912961986309523710.1128/iai.54.2.291-296.1986PMC260158KlineSEHedemarkLLDaviesSFOutbreak of tuberculosis among regular patrons of a neighborhood barNew England Journal of Medicine33342222271995779183810.1056/NEJM199507273330404LevetonCBarnassSChampionBLucasSDe SouzaBNicolMBanerjeeDRookGT-cell mediated protection of mice against virulentMycobacterium tuberculosis. Infection and Immunity5723903951989249225910.1128/iai.57.2.390-395.1989PMC313109MandrekarPCatalanoDGirouardLSzaboGHuman monocyte IL-10 production is increased by acute ethanol treatmentCytokine875675771996889143810.1006/cyto.1996.0076NelsonSBagbyGJBaintonBGSummerWRThe effects of acute and chronic alcoholism on tumor necrosis factor and the inflammatory responseJournal of Infectious Diseases16034224291989266842510.1093/infdis/160.3.422NelsonSMasonCBagbyGSummerWAlcohol, tumor necrosis factor, and tuberculosisAlcoholism: Clinical and Experimental Research1911724199510.1111/j.1530-0277.1995.tb01467.x7771645OrmeICollinsFProtection against *Mycobacterium tuberculosis* infection by adoptive immunotherapyJournal of Experimental Medicine15874831983660286110.1084/jem.158.1.74PMC2187069OrmeICollinsFAdoptive protection of the *Mycobacteria tuberculosis-*infected lungCellular Immunology841131201984642149210.1016/0008-8749(84)90082-0SzaboGVermaBCatalanoDSelective inhibition of antigen-specific T lymphocyte proliferation by acute ethanol exposure: The role of impaired monocyte antigen presentation capacity and mediator productionJournal of Leukocyte Biology5465345441993750404410.1002/jlb.54.6.534SzaboGGirouardLMandrekarPCatalanoDAcute ethanol treatment augments interleukin-12 production in activated human monocytesAnnals of the New York Academy of Sciences7954224251996895897310.1111/j.1749-6632.1996.tb52711.xVicente-GutierrezMMRuizADExtremeraBGGarciaJMBGeaFGLow serum levels of alpha-interferon, gamma-interferon, and interleukin-2 in alcoholic cirrhosisDigestive Diseases and Sciences369120912121991190994810.1007/BF01307510

1The terms “alcoholism” and “alcoholic” as used in this article are summary terms for the diagnoses of alcohol abuse and alcoholism.2Compared with *M. tuberculosis*, MAC is a less invasive infectious agent, but it has been associated with both pulmonary and disseminated disease in people with compromised immune systems (e.g., alcoholics or people with AIDS), just as TB has. The pathology and cell-mediated immune mechanisms involved in the body’s defense against MAC are similar to those seen in *M. tuberculosis* infection, making research on this agent relevant to an understanding of TB control.

Alcohol-induced overproduction of oxygen radicals in the liver, in contrast, may contribute to the development of alcoholic liver damage. In rats that received alcohol infusions for 1, 3, or 5 hours, for example, the Kupffer cells in the liver produced and secreted increased levels of superoxide anions, whether or not the cells were activated by contact with pathogens. Together, these observations imply that alcohol may have a dual negative effect on the body’s oxygen-radical production. First, alcohol may inhibit oxygen-radical and nitric oxide production in macrophages in the lung, where these substances are essential for killing microorganisms. Second, alcohol may increase oxygen-radical production in the liver, where these molecules may cause tissue damage.

### Alcohol’s Effects on Immunity

#### Effects on T Cells

Alcoholics and laboratory animals chronically ingesting alcohol have lower-than-normal numbers of all subpopulations of T cells in the blood, in the thymus—the gland where T cells mature—and in the spleen, where immune reactions are initiated. The mechanism underlying the alcohol-induced decrease in T-cell numbers still is unknown. Some researchers have suggested that acute alcohol exposure induces programmed cell death, or apoptosis, in immature T cells in the thymus. Acute alcohol exposure also results in increased apoptosis of mature lymphocytes and monocytes in the blood.

Alcohol also may reduce the ability of lymphocytes to proliferate and differentiate adequately after they have been activated by an antigen. Moreover, the number and function of delayed-type hypersensitivity T cells is reduced in alcoholics. As a result, the immune response to certain antigens and infections is depressed. How alcohol affects T-cell proliferation is not well understood. In chronically alcohol-fed rats, the T cells fail to proliferate adequately in response to stimulation by IL-2. The results of other investigations imply that decreased T-cell proliferation may be a consequence of the impaired function of accessory cells (e.g., antigen-presenting cells) after alcohol use. For example, the interaction of T cells with antigen-presenting monocytes or macrophages requires the presence of several proteins on the surfaces of both the T cells and the antigen-presenting cells (e.g., T-cell receptors and MHC molecules). The production of some of these proteins also is altered in alcohol-exposed cells. Finally, reduced T-cell proliferation may be attributed to the increased production of immunoregulatory cytokines (e.g., IL-10 and TGF-β) caused by alcohol.

Overall, the effects of both acute and chronic alcohol exposure result in a weakened cell-mediated immune response. Several diseases are characterized by a reduction in the cell-mediated immunity and a concomitant increase in the humoral immunity. Similarly, the immunological abnormalities observed after both chronic and acute alcohol consumption appear to be consistent with a decreased cell-mediated immunity characterized by reduced T-cell proliferation, accompanied by an enhanced humoral immunity marked by increased antibody levels. This shift in the immune response likely impairs the body’s defense against bacterial infections requiring a predominantly cell-mediated immune response, such as infections with *M. tuberculosis* or *Listeria monocytogenes*, which are discussed in the section “Consequences of Alcohol’s Effects on the Immune System.” Alcohol’s effects on the antibody-producing B cells is discussed in more detail in the following section.

#### Effects on B Cells

A characteristic immune-system aberration observed in alcoholics is the elevation of antibody levels in the blood. Similarly, acute alcohol consumption in mice increased antibody production in response to certain chemical substances. Because antibodies are produced by B cells, these observations indicate that alcohol alters either the number or function of B cells. To date, conflicting results exist regarding these two alternatives. Thus, clinical studies in humans found that the absolute number of B cells did not differ between alcoholics and non-alcoholics, but that B-cell functioning appeared to be altered in alcoholics. In contrast, in mice that had been fed alcohol for 14 days, the number of B cells in the spleen had decreased fivefold. Equally contradictory were the findings regarding B-cell functioning: Whereas clinical studies demonstrated elevated antibody levels in alcoholics, tissue-culture experiments to investigate these observations found that alcohol inhibited the B-cells’ antibody secretion.

One possible explanation for these conflicting findings is that alcohol interferes only with some aspects of B-cell functioning. For example, B cells do not respond to all antigens in the same manner. In response to some antigens, B cells require the assistance of cytokines secreted by T cells (i.e., T-cell–dependent responses), whereas in response to other antigens, T-cell activation is not required (i.e., T-cell–independent responses). Alcohol appears to affect these responses differently, because B cells in the spleens of alcohol-consuming animals showed impaired proliferation during a T-cell–dependent response but normal proliferation during a T-cell–independent response. Similarly, alcoholics exhibited an intact T-cell–independent antibody response after administration of a specific antigen. Thus, alcohol may interfere with antibody production indirectly by inhibiting the production of certain T-cell–derived cytokines required for B-cell function. The complexity of alcohol’s effects on B cells is underscored further by findings that alcohol impairs B-cell proliferation in response to the T-cell–derived cytokine IL-4 but not in response to the T-cell-derived cytokine IL-2.

#### Effects on Natural Killer Cells

NK cells are a type of white blood cell involved in the destruction of virus-infected and cancerous cells. Consequently, NK cells play an important role in preventing tumor development. Chronic alcohol consumption is associated with increased incidence of tumors, suggesting that NK cell activity may be impaired. In laboratory animals, chronic alcohol administration reduced the number and activity of NK cells. Tissue-culture experiments, in contrast, produced conflicting results, demonstrating that alcohol had either an inhibitory effect or no effect on NK cell activity. Finally, acute alcohol consumption temporarily reduced the ability of rats to eliminate certain tumor cells and prevent the development of tumor metastases.

#### Effects on Cytokines

Cytokines produced by lymphocytes (i.e., lymphokines, such as IL’s and interferons [IFN’s]) regulate the functions of immune cells as well as nonimmune cells (e.g., nerve cells and cells of hormone-producing organs). The effects of either chronic or acute alcohol use on cytokine production and function, however, are only partially understood. IL-2 is one of the most important T-cell–produced cytokines; it promotes the proliferation and survival of certain T-cell subpopulations. Although alcohol in tissue culture experiments had no effect on the ability of T cells to produce IL-2, it likely interferes with the T-cell response to IL-2. The potential intracellular mechanisms underlying these effects, however, remain unknown.

Alcoholics exhibit decreased blood levels of IFN-α, IFN-γ, and IL-2.^3^ In particular, the reduction of IFN-γ levels may be a key element underlying many of the immune alterations observed in alcoholics because this cytokine, in concert with the macrophage-derived IL-12, is crucial for induction of the cell-mediated immune response. Consequently, in the absence of appropriate IFN-γ stimulation in alcoholics, a preferential induction of the humoral immune response could occur. The accompanying lack of an appropriate cell-mediated immune response would make the alcoholics more susceptible to infections that require a T-cell response. Furthermore, decreased IFN-γ levels likely contribute to additional cytokine abnormalities (e.g., altered IL-12 levels), thereby further impairing the cell-mediated immune response.

## Consequences of Alcohol’s Effects on the Immune System

### Increased Susceptibility to Bacterial Infections

Alcoholics are considered “immuno-compromised hosts” because the incidence and severity of infections are increased in these patients. Infections with pathogens that reside within the host’s cells and cause diseases such as pneumonia or tuberculosis are especially prevalent. Thus, alcoholics have an increased incidence of pneumococcal pneumonia compared with the general population, and despite the use of antibiotics, the mortality among these patients remains disturbingly high (15 to 77 percent). Researchers have used mice to study some of the mechanisms underlying the increased susceptibility to infections by infecting the animals with *Listeria monocytogenes*, a bacterium that among other symptoms, causes liver damage in the animals. Mice that received an alcohol-containing diet for 7 days before being infected with *Listeria* developed greater liver damage than control animals that had received no alcohol. Although the alcohol treatment did not impair the migration of phagocytes to the liver, it did impair the animals’ ability to inhibit *Listeria* growth. Thus, even alcohol-fed mice that should have been able to stave off the infection, because they had previously been immunized with *Listeria*, had 100 times more *Listeria* organisms in their livers than did nonalcohol-treated controls. The body’s anti-*Listeria* defense is largely T-cell dependent and requires interactions between specific antigens, T cells, and phagocytic cells as well as IFN-γ and IL-12 induction. However, the exact roles of alcohol-induced aberrations in immune-cell interactions, antigen-presenting cell function, and IFN-γ and/or IL-12 production remain to be determined.

Alcohol use also impairs the body’s defense against pathogens infecting the lungs, such as pneumonia-causing bacteria (e.g., pneumococci, *Klebsiella pneumoniae*, and *Legionella pneumophila*) and *M. tuberculosis*. For example, in rats infected with pneumococci, the animals’ susceptibility to lethal pneumonia increased if they received alcohol for 1 week before the infection. Moreover, the alcohol-fed rats experienced an increased spread of the pneumococci from the lungs through the bloodstream compared with non-alcohol-treated rats and also failed to eliminate the pneumococci from the blood. Other studies investigating alcohol’s effects on the susceptibility to infections with *Klebsiella pneumoniae* and *Legionella pneumophila* indicated that chronic alcohol treatment suppressed the production and/or function of neutrophils and macrophages. Moreover, treatment with a protein factor that stimulates neutrophil production ameliorated the alcohol-induced immunosuppression by recruiting more neutrophils to the lungs.

### Increased Susceptibility to HIV

The relationship between alcohol use and susceptibility to infections with the human immunodeficiency virus (HIV), which causes acquired immune deficiency syndrome (AIDS), is an actively evolving area of research. Although ample data are available on the immunological abnormalities caused by alcohol use and HIV infection, respectively, knowledge concerning their combined immunosuppressive effects is more limited. Some researchers have proposed that alcohol’s modulatory effects on the immune system may increase the risk of initial HIV infection as well as accelerate the infection’s progression. Although this hypothesis still awaits formal confirmation, several findings support alcohol’s proposed influence. For example, one study found that HIV multiplied faster in blood cells isolated from binge drinkers or subjects who had received an acute alcohol dose than in cells from people who had not been exposed to alcohol (Bagasra et al. 1996). Moreover, a case report of an HIV-infected person demonstrated that the HIV infection progressed rapidly and that the patient developed full-blown AIDS after initiating heavy alcohol use (Fong et al. 1994). Finally, a study of HIV-positive intravenous drug users found that whereas alcohol use did not affect the proportion in the blood of the T-cell subpopulation that is the target of HIV infection, alcohol use did correlate with increases in the proportion of another T-cell subpopulation several years after the initial infection (Crum et al. 1996). Although the clinical significance of this observation is still unclear, the findings suggest that alcohol consumption may exacerbate HIV-induced changes in the immune system.

Current knowledge strongly suggests that alcohol use—potentially both acute and chronic—can increase a person’s susceptibility to HIV infection and contribute to alterations in the immune system that may result in an accelerated progression of the infection. However, further research is needed to elucidate the mechanism by which alcohol may modulate the biology of HIV infection. In addition to its biological effects, alcohol use may increase the risk of HIV infection by modifying the drinker’s behavior. For example, factors such as increased risk taking and uninhibited sexual behavior, which are associated with both acute and chronic alcohol use, can contribute to an increased risk for HIV infection.

### Consequences of Traumatic Injuries

Traumatic injuries frequently are associated with severe suppression of the immune system, which can lead to overwhelming infections and may result in multiple organ failure and even death. Alcohol intoxication not only increases the risk of sustaining traumatic injuries (e.g., in motor vehicle accidents) but also may exacerbate trauma-induced immunosuppression. Thus, one study found that acutely intoxicated patients (i.e., those with blood alcohol levels greater than 0.2 percent) who had sustained severe abdominal injuries had a 2.6 times greater incidence of infections than did patients who had not consumed alcohol (Gentilello et al. 1993). Moreover, rats that had received alcohol before sustaining severe burn injuries exhibited a significantly impaired cell-mediated immunity accompanied by an increased humoral immunity (i.e., elevated antibody production). Such an alcohol-related modification of the immune system after sustaining a traumatic injury could increase the patient’s risk of infections and prolong the trauma-related suppression of the immune system.

The mechanism underlying trauma-induced immunosuppression has not yet been identified. However, the cytokine TNF-α clearly plays an important role in this process. Thus, acute alcohol consumption before sustaining a traumatic injury can affect a patient’s TNF-α production and immunosuppression after the injury as follows:

- Within 0 to 3 days after the injury, TNF-α production in the monocytes decreases in patients whose blood alcohol levels exceed 0.1 percent at emergency-room admission, but increases in patients who have not consumed alcohol before sustaining the injury.
- Later during the posttrauma period (i.e., more than 6 days after the injury), the monocytes of alcohol-consuming trauma patients produce higher TNF-α levels than the monocytes of non-alcohol-consuming trauma patients.
- The increase in TNF-α production coincides with the development of posttraumatic immunosuppression, suggesting that acute alcohol consumption before sustaining major injuries increases the severity of the immunosuppression.

## Conclusions and Future Directions

Numerous research efforts have confirmed that both acute and chronic alcohol use have profound regulatory effects on the immune system. Studies in laboratory animals and in humans have demonstrated that even acute, moderate alcohol consumption can impair the body’s defense against bacteria and viruses, although these effects are likely only transient. The clinical implications of such a transient immunodepression still need to be studied further. For certain types of infections (e.g., HIV and mycobacteria), however, the failure of an appropriate initial immune response to pathogens can have profound and potentially prolonged effects on the immune system and the drinker’s health.

Researchers and clinicians are gaining further insight into the complex mechanisms and consequences of immunosuppression in chronic alcoholics. It is important, however, to dissect the effects caused by the body’s chronic exposure to alcohol itself and the effects of other alcohol-related immunomodulatory conditions, such as malnutrition, vitamin deficiencies, and alcoholic liver disease. Moreover, a better understanding of the specific immune system alterations caused by chronic alcohol consumption is necessary for designing effective therapeutic approaches to ameliorating immunosuppression in chronic alcoholics.

Researchers also are investigating the mechanisms underlying the differential effects of chronic and acute alcohol use on the immune system. For example, the increased levels of inflammatory cytokines observed in alcoholics contrast with the decreased inflammatory response seen after acute alcohol treatment. Finally, additional research is needed to delineate some of the intracellular signaling events in immune cells that are affected by acute and chronic alcohol use in order to better understand alcohol’s regulatory effects on the complex interactions of the immune system.

## Footnotes

1 For a definition of this and other technical terms used throughout this article, see the central glossary, pp. 93–96.

2 The alcohol concentrations used in these experiments were 10 to 100 millimoles per liter (mmol/L). Concentrations of 25 mmol/L approximate blood alcohol levels of 0.1 percent, the legal limit for driving in many States. Concentrations of 100 mmol/L correspond to blood alcohol levels of approximately 0.4 percent, which are sometimes seen in alcoholics after acute alcohol consumption.

3 The discrepant observations that alcohol did not affect the cells’ ability to produce IL-2 in tissue culture experiments, whereas alcoholics show decreased IL-2 levels in the blood likely can be attributed to the fact that tissue culture experiments monitor the consequences of acute alcohol exposure, but the findings in alcoholics reflect the effects of chronic alcohol exposure. However, this hypothesis has not been investigated in detail.
